# A randomized trial of an intervention to improve resident-fellow teaching interactions on the wards

**DOI:** 10.1186/s12909-016-0796-9

**Published:** 2016-10-20

**Authors:** Shruti Gupta, Jehan Alladina, Kevin Heaton, Eli Miloslavsky

**Affiliations:** 1 Division of Nephrology, Department of Medicine, Massachusetts General Hospital, 55 Fruit Street, Boston, MA 02114 USA; 2 Division of Pulmonary and Critical Care Medicine, Department of Medicine, Massachusetts General Hospital, Boston, USA; 3 Department of Medicine, Massachusetts General Hospital, Harvard Medical School, Boston, USA; 4 Division of Rheumatology, Department of Medicine, Massachusetts General Hospital, Yawkey Center for Outpatient Care, Harvard Medical School, 55 Fruit Street, Suite 2C, Boston, MA 02114 USA

**Keywords:** Inpatient subspecialty consultation, Internal medicine, Fellows, Residents, Teaching interaction, Communication

## Abstract

**Background:**

Subspecialty fellows can serve as a tremendous educational resource to residents; however, there are multiple barriers to an effective resident-fellow teaching interaction in the setting of inpatient consultation. We designed and evaluated a resident-directed intervention to enhance communication and teaching during consultation on the general medicine wards.

**Methods:**

Five medical teams were randomized to receive the intervention over a 3 month period (3 control, 2 intervention teams). The intervention was evaluated with pre and post-intervention surveys.

**Results:**

Fifty-nine of 112 interns completed the pre-intervention survey, and 58 completed the post-intervention survey (53 % response rate). At baseline, 83 % of the interns noted that they had in-person interactions with fellows less than 50 % of the time. 81 % responded that they received teaching from fellows in less than 50 % of consultations. Following the intervention, the percentage of interns who had an in-person interaction with fellows greater than 50 % of the time increased in the intervention group (9 % control versus 30 % intervention, *p* = 0.05). Additionally, interns in the intervention group reported receiving teaching in more than 50 % of their interactions more frequently (19 % control versus 42 % intervention, *p* = 0.05). There were no differences in other measures of teaching and communication.

**Conclusions:**

We demonstrate that a time-efficient intervention increased perceptions of in-person communication and the number of teaching interactions between interns and fellows. Further studies are warranted to determine whether such an approach can impact resident learning and improve patient care.

**Electronic supplementary material:**

The online version of this article (doi:10.1186/s12909-016-0796-9) contains supplementary material, which is available to authorized users.

## Background

Graduate medical education training programs are faced with the challenge of developing trainee expertise despite increased work hour limitations. The majority of trainee learning takes place in the everyday clinical environment, making work-based learning opportunities increasingly valuable for trainee development [1, 2]. The role of consultation in the inpatient setting is expanding; [3–5] subspecialty consultation represents an important opportunity for resident work-based learning. Within academic medical centers, communication during consultation most frequently occurs between trainees [6], making the teaching interaction between residents and fellows important. Both fellows and residents have a strong interest in teaching and learning, respectively [7]. However, many barriers to teaching during consultation exist in the hospital environment [7–10]. Suboptimal communication of the consult question, fellow “pushback” on consult requests, lack of incentives for fellows to teach, perceptions among residents that fellows may not be interested in teaching, and among fellows that residents may not be interested in learning, may hinder the resident-fellow interaction.

Effective communication between consultants and the primary team is critical for patient care and forms the cornerstone for successful resident-fellow teaching interactions [7, 11, 12]. Several resident-directed interventions, such as the 5 Cs Model of Consultation (5Cs) [13], and the CONSULT card [14], have been proposed to improve communication between residents and subspecialty consult services as it relates to patient care. Of these, the 5Cs model has been the most rigorously studied, demonstrating that a resident-directed intervention can improve communication during consultation in the Emergency Department [10, 11]. However, no study has previously attempted to help trainees overcome barriers specifically to resident-fellow teaching interactions on the wards. A number of barriers to effective resident-fellow teaching interactions identified in the literature appear to be amenable to resident-directed intervention, such as communication of the consult question and perceptions of fellows and residents with respect to teaching and learning in this setting [7–9, 15]. Finally, while several fellow-directed interventions have focused on improving fellow teaching skills [16–20], fellow-directed interventions may be more challenging to implement because the intervention would need to be applied uniformly across many subspecialty divisions. The purpose of this study was to 1) design a resident-directed intervention to address the barriers to teaching in the setting of consultation which have been identified previously and 2) evaluate the intervention’s impact on teaching interactions on the general medicine wards.

## Methods

### Setting and participants

The intervention was developed by the investigators with input from key clinical faculty, Chief Residents and several residents not participating in the evaluation portion of the study.

The evaluation phase consisted of a two-group randomized study conducted at a single academic medical center in the United States over a 3 month period, from March to May. The core of the inpatient resident medical service at the institution consists of five similar general medicine teams (team A – team E). Each team is composed of 5 interns, 1–2 supervising residents and 2 attending physicians. Interns are postgraduate year 1 (PGY-1) residents, while the supervising residents are postgraduate year 2 (PGY-2) residents. Attending physicians had completed residency and oversaw the teams. Two teams were randomly assigned (using random number generation) to the intervention group (team C and team E) for the duration of the 3-month study, while the remaining three inpatient medicine teams served as the control group, yielding approximately a 2:3 allocation ratio. Because the teams have a similar structure and care for the same range of medical problems there was low potential for bias in assigning teams to the intervention. All interns, supervising residents, and attending physicians worked on their respective teams for 2–4 week blocks and were eligible to participate in the study. The study period consisted of 4 blocks on three teams and 5 blocks on two teams. We estimated that 29 participants in each group would be required to complete the study in order to yield an effect size of 0.75 with 80 % power. All study teams operated within a team-based care model in which interns cared for all 20–24 patients on a team, rather than an individual list of patients. Interns were primarily responsible for communication with fellows, who were defined as trainees in a subspecialty field of medicine.

### Intervention

The intervention was developed in order to address previously identified barriers to effective teaching interactions between residents and fellows [7–10]. Input from clinical faculty, residents not participating in the study and Chief Residents was sought during development of the intervention, which was then revised accordingly. The intervention was designed with the principles that 1) it must be time efficient, as residents and fellows both identified time limitations as a barrier to teaching and learning during consultation [7], 2) it could be implemented without a significant change to resident team workflow, and 3) it did not depend on fellow involvement to be successful because implementation across all fellowship programs could serve as a barrier to implementing the intervention. Based on these principles, we designed a 4-step intervention. Each of the four steps--and the rationale for inclusion of each step-- is described in Table 1.

**Table 1:** The 4 steps of an intervention designed to improve communication between residents and fellows

Action	Rationale
1. The supervising resident assists the intern in coming up with a specific consult question using a task-list software (Apprentice, Boston MA) used for patient care.	“Pushback” or reluctance to see the consult on the part of fellows has been cited as a major barrier to resident-fellow teaching interactions [7]. One of the most common reasons for fellow “pushback” is the lack of a clear consult question. Interns, due to their limited experience, can find it challenging to effectively determine the consult question [7]. This step of the intervention was designed to enhance the quality of the consult question posed to the consult team by the interns.
2. When requesting the consultation from a fellow (initial interaction, which typically occurred by telephone), interns should ask the fellow to discuss the case in-person after they have seen the patient (follow-up interaction). The supervising resident encourages interns to have an in-person interaction with the fellow at least once during the consult.	Teaching interactions most commonly occur during in-person communication (as compared to by telephone or consult note) [7]. Fellows are typically responsible for initiating in-person communication when relating their recommendations. This step of the intervention sought to incentivize interns to have in-person communication with fellows and incentivize fellows to locate the interns in order to have an in-person interaction.
3. During the follow-up interaction, interns encouraged to ask the fellow at least one question about the case in order to initiate the teaching interaction.	Fellows are more likely to teach residents that express an interest in learning [7]. This is in part due to the perception, on the part of fellows, that interns may be too busy to learn or not interested in learning [7]. Therefore, interns may be responsible for initiating the teaching interaction. This step of the intervention was designed to encourage interns to initiate the teaching interaction, thereby incentivizing fellows to teach.
4. Interns share a teaching point they learned from the fellow on rounds.	In the team-based care model employed in the general medicine service, this step of the intervention both incentivized the interns to initiate the teaching interaction and disseminated the teaching to the rest of the team.

The supervising residents on the intervention teams were introduced to the study and the intervention via an email communication. Intervention teams (which included the interns and the supervising residents) then underwent training on the intervention by one of the investigators at the start of each 2–4 week rotation. The supervising resident was responsible for promoting the implementation of the intervention on their team. The supervising resident assisted the interns in coming up with a consult question, reminded interns to have in-person interactions with fellows, and encouraged them to share teaching points gleaned from fellows during rounds. Midway through each rotation, one of the investigators communicated with the supervising resident on the intervention teams to ensure implementation. A poster describing the intervention was displayed in the team room of the intervention teams (intervention and control teams did not share a workspace). Control teams were informed that investigators were conducting a study examining resident-fellow interactions, but they did not receive any intervention training, and posters were not displayed in their team rooms.

Fellows were made aware of the intervention via an email, but were not aware of team assignments. Fellows were specifically not included in the intervention because the consult requests they receive from Internal Medicine services constitute only a portion of the total number of consults in the hospital. In addition, the goal was for fellows to remain unbiased in their interactions with all teams, both control and intervention. Attending physicians were notified about the study but were not formally trained in the intervention, which was designed to be implemented predominantly by the supervising residents. Attending physician and supervising residents on all teams were surveyed in order to evaluate their perceptions of intern interactions with fellows. The study was approved by the Partners Institutional Review Board.

### Evaluation instruments

Both the intervention and control groups were administered anonymous, identical pre-intervention surveys focusing on perceptions of communication and teaching during consultation (Additional file 1). Survey answer choices were both quantitative (e.g. what proportion of direct communications occurred in-person: none, less than 25 %, 25–49 %, 50–74 %, 75–100 %) and qualitative (e.g. rate your level of comfort asking fellows questions: 5-point Likert scale --very uncomfortable to very comfortable).

The intervention was evaluated with post-intervention surveys, which utilized the same questions as the pre-intervention survey (Additional file 1). Several additional questions assessing perceptions of the intervention were included in the post-intervention surveys administered to the intervention teams. The surveys were developed by the authors based on the consultative medicine literature, reviewed by two content experts, cognitively tested with residents not participating in the study, and subsequently revised by the authors.

### Statistics

Survey responses were dichotomized for analysis (Additional file 2). The two categories consisted of the three lowest ratings and the two highest ratings; these generally corresponded to “less than 50 % of the time” versus “more than 50 % of the time,”“uncomfortable” versus “comfortable,” etc., depending on the question stem. The control group pre-rotation responses were compared to the intervention group pre-rotation responses. Similarly, the control group post-rotation survey responses were compared to the intervention group post-rotation responses. Differences between groups were compared using the Chi-square or Fisher exact test as appropriate, with two-tailed tests of significance (alpha < 0.05). JMP Pro v11.0 (Cary, NC) was used for analysis.

## Results

### Intern pre-intervention survey results

Fifty-nine of 112 participating interns completed the pre-intervention survey (30 in the control group and 29 in the intervention group, 53 % response rate) (Table 2). There were no baseline differences between the two groups. Overall, eighty-three percent of interns reported that in-person interactions with fellows occurred less than 50 % of the time on the day of initial consultation and 81 % noted receiving teaching from fellows in less than 50 % of initial consultations. Forty-three percent of interns felt that they received “pushback” from fellows during at least a quarter of their consult requests. Only 32 % rated the overall communication between their team and consultants as either “very good” or “excellent.”

**Table 2:** Pre-Intervention intern survey

	Control		Intervention		*P*-value
	*N* = 30		*N* = 29		
	Yes	No	Yes	No	
Direct communication ≥50 % of the time	18 (60 %)	12 (40 %)	23 (79 %)	6 (21 %)	0.11
In-person communication ≥50 % of interactions	4 (13 %)	26 (87 %)	6 (21 %)	23 (79 %)	0.51
Frequency of teaching ≥ 50 % of interactions	4 (13 %)	26 (87 %)	7 (25 %)	22 (75 %)	0.33
Initiate interaction >50 % of the time	17 (57 %)	13 (43 %)	12 (41 %)	17 (59 %)	0.24
Learn new facts >50 % of the time	16 (53 %)	14 (47 %)	17 (59 %)	12 (41 %)	0.68
Amount of teaching (just the right amount or more)	8 (27 %)	22 (73 %)	7 (24 %)	22 (76 %)	0.82
Share teaching points with the team (often, most of the time)	17 (57 %)	13 (43 %)	18 (62 %)	11 (38 %)	0.67
Find sharing of teaching points with team helpful (agree or strongly agree)	28 (97 %)	2 (3 %)	28 (97 %)	1 (3 %)	1.00
Pushback in <15 % of consults	14 (47 %)	16 (53 %)	11 (38 %)	18 (62 %)	0.50
Comfort asking questions (comfortable, very comfortable)	17 (57 %)	13 (43 %)	17 (59 %)	12 (41 %)	0.88
Overall communication (very good, excellent)	6 (20 %)	24 (80 %)	8 (29 %)	21 (71 %)	0.45

### Intern post-intervention survey results

Fifty-eight of 112 interns completed the post-intervention survey (32 in the control group, 26 in the intervention group, 52 % response rate) (Table 3). More interns had an in-person interaction with fellows during more than 50 % of the initial consult interactions in the intervention group (30 % in the intervention group versus 9 % in the control group, *p* = 0.05). Additionally, more interns in the intervention group reported receiving teaching in more than 50 % of their interactions (42 % in the intervention group versus 19 % in the control group, *p* = 0.05). There were no differences in the amount of perceived “pushback” from fellows, overall teaching from fellows, ratings of overall communication, or the frequency of sharing teaching points on rounds as a result of the intervention.

**Table 3:** Post-Intervention intern survey

	Control		Intervention		*P*-value
	*N* = 32		*N* = 26		
Direct communication >50 % of the time	18 (56 %)	14 (44 %)	19 (73 %)	7 (27 %)	0.18
In-person communication >50 % of interactions	3 (9 %)	29 (91 %)	8 (31 %)	18 (69 %)	0.05
Frequency of teaching ≥ 50 % of interactions	6 (19 %)	26 (71 %)	11 (42 %)	15 (58 %)	0.05
Initiate interaction >50 % of the time	13 (41 %)	19 (59 %)	11 (42 %)	15 (58 %)	0.90
Learn new facts >50 % of the time	17 (53 %)	15 (47 %)	16 (62 %)	10 (38 %)	0.52
Amount of teaching (just the right amount or more)	12 (38 %)	20 (62 %)	9 (35 %)	17 (65 %)	0.82
Share teaching points with the team (often, most of the time)	17 (55 %)	15 (45 %)	15 (58 %)	11 (42 %)	0.83
Find sharing of teaching points with team helpful (agree or strongly agree)	30 (94 %)	2 (6 %)	26 (100 %)	0 (0 %)	0.50
Pushback in <15 % of consults	11 (34 %)	21 (66 %)	7 (27 %)	19 (73 %)	0.54
Comfort asking questions (comfortable, very comfortable)	22 (69 %)	10 (31 %)	23 (88 %)	3 (12 %)	0.11
Overall communication (very good, excellent)	11 (34 %)	21 (66 %)	13 (50 %)	13 (50 %)	0.23

### Supervising resident and attending survey results

Sixty-two supervising residents and attending physicians completed the pre-intervention survey and 60 completed the post-intervention survey (65 % response rate). There were no differences between intervention and control team supervising residents or attending physicians (data not shown). Analysis of combined pre and post intervention data showed that attending physicians and supervising residents rated overall communication between their team and the consulting teams more favorably than interns (Table 4).

**Table 4:** Rating of overall communication with consulting teams

	Poor, fair, good	Very good, excellent	*P*-value
Interns	78 (67 %)	38 (33 %)	0.02
Supervising residents	21 (46 %)	25 (54 %)	
Attendings	40 (53 %)	36 (47 %)	

### Implementation and perception of the intervention

Of the intervention teams, 76 % of interns and 77 % of supervising residents reported that the intervention improved learning by more than “a little” (3–5 on Likert scale) (Table 5). Fifty-six percent of interns and 78 % of supervising residents reported an improvement in overall communication with the consulting teams as a result of the intervention by more than “a little” (3–5 on Likert scale). Although the vast majority of interns on the intervention teams carried out the intervention at least “some of the time,” implementation was not uniform, with only 40 % of interns noting that they implemented the intervention “most” or “all of the time.”

**Table 5:** Improvement in learning and communication as a result of the Intervention

		Not at all, a little bit	Some, quite a bit, a lot	Not sure
Intervention improved communication	Interns	4 (16 %)	19 (76 %)	2 (8 %)
	Supervising residents	2 (22 %)	7 (77 %)	0 (0 %)
Intervention improved learning	Interns	7 (28 %)	14 (56 %)	4 (16 %)
	Supervising residents	1 (22 %)	7 (78 %)	1 (22 %)

## Discussion

Restricted work hours and increasing clinical obligations may limit dedicated time for education during residency training; as a result, work-based learning is becoming increasingly important [1, 2]. The resident-fellow interaction in the setting of consultation represents an important opportunity to augment work-based teaching encounters. We demonstrate that a time-efficient intervention improved the perceived frequency of in-person communication and teaching encounters between interns and fellows on the general medicine wards.

Previous interventions aimed at improving communication between primary and consulting teams, such as the 5Cs model, have effectively addressed how to communicate the key portions of the consult; however, no resident-directed intervention to date have focused on enhancing the resident-fellow teaching interaction or overcoming the barriers to teaching that arise in the hospital environment. Indeed, our study showed that of all interns surveyed, only 32 % rated the overall communication between their team and consultants as either “very good” or “excellent” at baseline. Our intervention was focused on helping residents and fellows surmount the previously described barriers to teaching during consultation by encouraging residents to communicate an effective consult question, promote in-person communication during the follow-up phase of the interaction (when the fellow communicates recommendations to the primary team), and alter the fellows’ perceptions that residents may be too busy or uninterested in engaging in teaching interactions.

Clear communication of a specific consult question has been identified in multiple studies as a key component of effective consultation and is addressed in the 5Cs model [11–13]. However, multiple barriers limit trainees’ ability to determine and communicate a specific question consistently [7–10]. Such barriers include limited knowledge of the patient’s clinical course (e.g. if the intern did not admit the patient), insufficient medical knowledge (which can impact the trainee’s ability to communicate an effective question), or disagreement with the consult request (e.g. when the request for consultation comes from a more senior member of the team, such as the attending physician) [7, 21, 22]. The first step of our intervention was designed to address this barrier by encouraging interns and supervising residents to establish a precise question prior to requesting the consult. Communication of an effective consult question can limit “pushback” by fellows on resident consult requests, thus leading to more positive interactions later in the consultation process [7].

Teaching is most likely to occur during in-person interactions between residents and fellows, rather than during a telephone conversation or through a consultation note [7]. Patient care also likely benefits from direct, in-person communication between the fellow and the primary team [1, 2, 7, 8]. However, residents and fellows may not have the opportunity to communicate in-person due to barriers that arise in the hospital environment, such as time constraints, disparate resident and fellow rounding schedules, and the physical location of residents in the hospital (e.g. if residents are not located near their patients on non-regionalized medical teams) [7]. Indeed, the vast majority of interns reported that face-to-face interactions with fellows on the initial day of consultation occur less than half of the time. Our intervention addressed this barrier by encouraging interns to “invite” fellows to relay their consult recommendations in-person. This step was designed to not only incentivize fellows to have an in-person interaction, but to alert them that the resident was interested in their recommendations and engaged in the consultation process. We found that more interns on the intervention teams had in-person interactions with fellows on a regular basis.

Traditionally, teaching interactions have been initiated and directed by the teacher rather than the learner. However, given time constraints and other barriers that impact the resident-fellow interaction, fellows are more likely to teach residents who demonstrate an interest in learning from the fellow [7]. Our intervention was designed to encourage interns to engage fellows in teaching interactions by asking them a question about the case. Our prior work demonstrated that this is an effective method to promote both resident and fellow engagement in teaching and learning [7]. In fact, the majority of interns in the intervention group reported that the intervention improved learning and communication with the consulting teams. In addition, we aimed to promote increased communication between the intern and fellow, which may lead to the development of familiarity. Familiarity and collegiality have been postulated to facilitate the resident-fellow interaction and therefore may subsequently enhance the teaching interaction [7, 8]. It is notable, for instance, that supervising residents and attendings rated communication between consultants and interns more positively, suggesting that familiarity and/or the level of clinical experience may influence consult communication.

Despite an increase in the perceived frequency of teaching interactions, the perceived “amount” of teaching did not increase. While this may have been due to the limitations of our survey instrument, another possibility is that fellows may not have been able to consistently teach in an effective manner. Teaching in the setting of consultation can be quite challenging because the fellow must assess the learner, determine teaching objectives, and deliver useful teaching points in a limited amount of time (typically, in our experience, a resident and fellow have less than ten minutes to engage in a teaching interaction). Furthermore, the lack of a longitudinal relationship between the resident and the fellow typically makes learner assessment even more challenging. The nature of the interaction differs significantly from teaching opportunities fellows may have experienced as residents, which may have taken the form of delivering a lecture, teaching in a small group setting, or leading a medical team. Training programs that are therefore designed to improve fellows’ teaching skills--particularly in the areas of learner-centered teaching and rapid learner assessment and feedback-- have significant opportunities to improve resident-fellow teaching interactions [16–20, 23].

It is notable that the amount of direct communication occurring more than 50 % of the time decreased over the course of the study. This was surprising, as we had expected the control group to seek out more teaching interactions from consultants due to the maturation effect [24]. One possible explanation is that teaching interactions decrease in the second half of the academic year, which is when this study took place. We hypothesize that this change may be secondary to resident and/or fellow burn-out or prior negative interactions between residents and fellows; however, this requires further study for confirmation. The amount of perceived “pushback” also did not change despite an effort to communicate a clear and specific question. It is possible that “pushback” is affected by factors outside of the consult question [7]. For example, as previously noted, interns rated communication with consultants lower than supervising residents and attending physicians, suggesting that the level of training may affect perceived interactions with consultants. It is notable that perception of overall communication did not improve as a result of the intervention. This could be attributed to incomplete implementation of the intervention, potentially due to limited training on the intervention as well as workload and time constraints faced by both residents and fellows. Furthermore, hospital culture and ingrained habits, such as relying on pages and telephone conversations, can be difficult to change. It is possible that more extensive training or implementing the intervention during the beginning of the academic year could have made the intervention more effective. Actively involving fellows might have also made an additional impact.

There are several notable limitations in our study. The survey response rate in the study was modest at 53 %, which may have affected the study results and allowed for possible selection bias in the study. Furthermore, an intention to treat analysis was not feasible given such a response rate. We allowed for a relatively short period of time for survey completion in order to minimize recall bias, which may have limited the response rate. Moreover, conducting the study at the end of the academic year and during a busy inpatient rotation may have also had an impact as both survey fatigue and clinical workload may have reduced the response rate. Introducing incentives for participation and allocating time at the end of the rotation to complete the survey are methods that could increase the response rate in future studies.

Our study had several additional limitations. The participants in the intervention group were aware of the experiment, leading to the possibility of participant bias. In addition, because the surveys were anonymous, we could not perform a matched pairs analysis or determine the degree of overlap between the residents who completed the pre and post surveys in the control and intervention groups. As described previously, there was incomplete implementation of the intervention, with only 40 % of the interns reporting implementing it “most” or “all of the time.” Furthermore, the study may have therefore been underpowered to detect smaller differences. Due to a lack of previous data on this subject, our power calculation was based on estimates. Our study may not be generalizable to attending-trainee interactions or to institutions with fewer trainees where there is more familiarity between residents and fellows. Moreover, the team-based care model employed on our general medicine service adds additional challenges to the consult interaction, possibly limiting the effect of our intervention. Further study of this intervention in a traditional patient care model is warranted. We could not investigate potential differences in communication and teaching between various consult services as there was no data comparing the teaching quality of fellows from one subspecialty against another, and the number of consultations was not quantified. While our anecdotal experience suggested that the intervention was time-efficient, we did not measure the average time required to implement the intervention. Finally, our survey-based study was based on subjective findings and participants’ perception; more objective measurements could be considered in future studies.

## Conclusions

In summary, our time-efficient, four-step intervention increased perceptions of in-person communication and the number of teaching interactions between interns and fellows on the general medicine wards. Enhanced resident-fellow teaching interactions may have a far-reaching effect by not only improving trainee knowledge, but also improving patient care through enhanced communication between teams, as well as improving subspecialty fellows’ knowledge and clinical skills through teaching. Further studies are warranted to determine whether similar approaches can impact resident learning and improve patient care by enhancing communication and collaboration between consultants and the primary team.

## Additional files

Additional file 1: The pre and post surveys distributed to participants. (DOCX 20 kb)
Additional file 2: Data from pre and post surveys among control and intervention groups. (XLSX 13 kb)
