# Supplementary material for: A randomized trial of an intervention to improve resident-fellow teaching interactions on the wards
Source: BMC Med Educ. 2016 Oct 20;16:276. doi: 10.1186/s12909-016-0796-9 (PMC5072305; doi:10.1186/s12909-016-0796-9)
Supplement: Additional file 1: — The pre and post surveys distributed to participants. (DOCX 20 kb) [file 12909_2016_796_MOESM1_ESM.docx]

**Intern Pre-Rotation Survey**

This survey pertains to your interactions with Internal Medicine subspecialty fellows (Allergy, Cardiology, Endocrinology, GI, Hematology/Oncology, ID, Palliative Care, Pulmonary, Renal, Rheumatology) **over the past academic year**.

1. As the responding clinician, how often do you have an in-person or telephone communication with the consulting fellow regarding the initial consult recommendations?
   1. Never
   2. Less than 25%
   3. 25-49%
   4. 50-74%
   5. 75-100%
2. Of the above direct communications (in-person or telephone), what proportion occur in-person?
   1. Never
   2. Less than 25%
   3. 25-49%
   4. 50-74%
   5. 75-100%
3. In what proportion of consults do you receive teaching from the fellow? (teaching includes the fellow sharing their thought process, teaching a general approach to the patient, teaching procedures or teaching factual knowledge)
   1. Never
   2. Less than 25%
   3. 25-49%
   4. 50-74%
   5. 75-100%
4. When you receive teaching from a fellow, what proportion of the teaching interactions do you initiate?
   1. Never
   2. Less than 25%
   3. 25-49%
   4. 50-74%
   5. 75-100%
5. In what proportion of consults do you learn one or more new facts or concepts from your interaction with the fellow?
   1. 75-100%
   2. 50-74%
   3. 25-49%
   4. Less than 25%
   5. None
6. Please rate the amount of teaching you receive from fellows when they are consulting on your patients.
   1. Very little
   2. Less than I’d like
   3. Just the right amount
   4. More than I’d like
   5. Way too much
7. How often do you share teaching points you learned from consults with your team during rounds?
   1. Never
   2. Rarely
   3. Sometimes
   4. Most of the time
   5. Always
8. I find it helpful when others share what they learned from fellows during rounds.
   1. Strongly disagree
   2. Disagree
   3. Neutral
   4. Agree
   5. Strongly agree
9. When calling a new consult, how often do you experience pushback from the fellows?
   1. >50%
   2. 25-49%
   3. 15-24%
   4. 5-14%
   5. Less than 5%
10. Please rate your level of comfort asking fellows questions or clarifying their recommendations.
    1. Very uncomfortable
    2. Uncomfortable
    3. Neutral
    4. Comfortable
    5. Very comfortable

**Junior Resident/Attending Pre-Rotation Survey**

This survey pertains to your interactions with Internal Medicine subspecialty consult services (Allergy, Cardiology, Endocrinology, GI, Hematology/Oncology, ID, Pulmonary, Renal, Rheumatology) **during your previous Bigelow rotations.**

1. Please rate the communication between your Bigelow teams and the medicine subspecialty consult services. (communication relates to understanding the consultants’ plan and thought process, as well as answering your questions in a timely manner).
   1. Excellent
   2. Very Good
   3. Good
   4. Fair
   5. Poor
2. How often are teaching points from interactions with consultants discussed on rounds (sign out or morning rounds)?
   1. Always
   2. Most of the time
   3. Sometimes
   4. Rarely
   5. Never

**Intern Post-Rotation Survey**

This survey pertains to your interactions with Internal Medicine subspecialty fellows (Allergy, Cardiology, Endocrinology, GI, Heme/Onc, ID, Palliative Care, Pulmonary, Renal, Rheumatology) **during this Bigelow rotation**.

1. As the responding clinician, how often did you have an in-person or telephone communication with the consulting fellow when they are giving you the initial consult recommendations?
   1. Never
   2. Rarely (approximately < 25%)
   3. Sometimes (approximately 25-50%)
   4. Often (approximately 50-75%)
   5. Most of the time (approximately 75-100%)
2. Of the above direct communications (in-person or telephone), how often did they occur in-person?
   1. Never
   2. Rarely (approximately < 25%)
   3. Sometimes (approximately 25-50%)
   4. Often (approximately 50-75%)
   5. Most of the time (approximately 75-100%)
3. This rotation, during what proportion of consults did you receive teaching from the fellow? (teaching includes the fellow sharing their thought process, teaching a general approach to the patient, teaching procedures or teaching factual knowledge)
   1. Never
   2. Rarely (approximately < 25%)
   3. Sometimes (approximately 25-50%)
   4. Often (approximately 50-75%)
   5. Most of the time (approximately 75-100%)
4. When you received teaching from a fellow during this rotation, how often did you initiate the teaching interaction?
   1. Never
   2. Rarely (approximately < 25%)
   3. Sometimes (approximately 25-50%)
   4. Often (approximately 50-75%)
   5. Most of the time (approximately 75-100%)
5. This rotation, how often did you learn one or more new facts or concepts from your interaction with the fellow?
   1. Never
   2. Rarely (approximately < 25%)
   3. Sometimes (approximately 25-50%)
   4. Often (approximately 50-75%)
   5. Most of the time (approximately 75-100%)
6. This rotation, please rate the amount of teaching you receive from fellows when they are consulting on your patients.
   1. Far too little
   2. Too little
   3. About right
   4. Too much
   5. Far too much
7. This rotation, how often did you share teaching points you learned from consults with your team during rounds or through Apprentice?
   1. Never
   2. Rarely
   3. Sometimes
   4. Often
   5. Most of the time
8. I find it helpful when others shared what they learned from fellows.
   1. Strongly disagree
   2. Disagree
   3. Neutral
   4. Agree
   5. Strongly agree
9. When calling a new consult during this rotation, how often did you experience pushback from the fellows?
   1. Never
   2. Rarely (approximately < 10%)
   3. Occasionally (approximately 10-25%)
   4. Often (approximately 25-50%)
   5. Very often (approximately >50%)
10. This rotation, please rate your level of comfort asking fellows questions or clarifying their recommendations.
    1. Very uncomfortable
    2. Uncomfortable
    3. Neutral
    4. Comfortable
    5. Very comfortable
11. This rotation, please rate the communication between your Bigelow team and medicine subspecialty consult services. (communication relates to understanding the consultants’ plan and thought process, as well as answering your questions in a timely manner)
    1. Poor
    2. Fair
    3. Good
    4. Very Good
    5. Excellent
12. Please rate your overall experience on this Bigelow rotation
    1. Poor
    2. Fair
    3. Good
    4. Very Good
    5. Excellent

***Note: Questions 13-16 were added for the intervention group***

1. To what extent were you able to implement the fellow intervention during this Bigelow rotation?
   1. Not at all
   2. A little bit
   3. Some of the time
   4. Most of the time
   5. All of the time
   6. Not sure
2. To what extent did the fellow intervention improve your learning during this Bigelow rotation?
   1. Not at all
   2. A little bit
   3. Some
   4. Quite a bit
   5. A lot
   6. Not sure
3. To what extent did the fellow intervention improve your interaction with medicine subspecialty fellows during this Bigelow rotation?
   1. Not at all
   2. A little bit
   3. Some
   4. Quite a bit
   5. A lot
   6. Not sure
4. We would appreciate any comments you have on the intervention, its implementation or interactions with fellows in general below.

**Junior/Attending Resident Post-Rotation Survey:**

This survey pertains to your interactions with Internal Medicine subspecialty consult services (Allergy, Cardiology, Endocrinology, GI, Hematology/Oncology, ID, Pulmonary, Renal, Rheumatology) **during this Bigelow rotation.**

1. Please rate the communication between your Bigelow team and the medicine subspecialty consult services. (communication relates to understanding the consultants’ plan and thought process, as well as answering your questions in a timely manner)
   1. Excellent
   2. Very Good
   3. Good
   4. Fair
   5. Poor
2. How often were teaching points from interactions with consultants discussed on rounds (sign out or morning rounds)
   1. Always
   2. Most of the time
   3. Sometimes
   4. Rarely
   5. Never
3. Please rate your overall experience on this Bigelow rotation
   1. Poor
   2. Fair
   3. Good
   4. Very Good
   5. Excellent

***Note: Questions 4-7 were added for the intervention group***

1. To what extent was your team able to implement the fellow intervention during this Bigelow rotation?
   1. Not at all
   2. A little bit
   3. Somewhat
   4. Almost completely
   5. Completely
   6. Not sure
2. To what extent did the fellow intervention improve your team’s learning during this Bigelow rotation?
   1. Not at all
   2. A little bit
   3. Some
   4. Quite a bit
   5. A lot
   6. Not sure
3. To what extent did the fellow intervention improve your team’s interaction with medicine subspecialty fellows during this Bigelow rotation?
   1. Not at all
   2. A little bit
   3. Some
   4. Quite a bit
   5. A lot
   6. Not sure
4. We would appreciate any comments you have on the intervention, its implementation or interactions with fellows in general below.
